# Analysis of *Staphylococcus aureus* Molecules in Non-Treated Blood Using Mercury Immobilized Carbon Nanotube Sensor

**DOI:** 10.3390/molecules27061837

**Published:** 2022-03-11

**Authors:** In Hea Cho, Kwang Jin Choi, Ji Hyun Kim, Kyung Lee, Suw Young Ly

**Affiliations:** 1 Department of Anesthesiology and Pain Medicine, Korea University Anam Hospital, Seoul 02841, Korea; donkeywife@naver.com; 2 Smith College of Liberal Arts, Sahmyook University, 815, Hwarang-ro, Nowon-gu, Seoul 01795, Korea; kjchoi@syu.ac.kr; 3 College of Nursing, Sahmyook University, 815, Hwarang-ro, Nowon-gu, Seoul 01795, Korea; kimjh@syu.ac.kr; 4 Biosensor Research Institute, Seoul National University of Science and Technology, Seoul 01811, Korea; paster0228@hanmail.net

**Keywords:** *Staphylococcus aureus*, molecular probe, voltammetry, assay, carbon nanotube sensor, mercury immobilization

## Abstract

Staphylococcus aureus bacteria is a ubiquitous Gram-positive microorganism that causes infections related to the sudden infant death syndrome. Recently, basic detection methods depend on complicated PCR amplification, electric separation, spectric adsorption and other detection systems. However, in this study, simplified sensitive voltammetric skills are developed. To identify an effective diagnostic method for Staphylococcus aureus (SA), a voltammetric sensing probe was sought using mercury immobilized on a carbon nanotube sensor (MCN). The voltammetric MCN conditions were optimized through stripping and cyclic voltammetry. Diagnostic electrolyte was used on non-treated blood sera as an electrolyte solution. The optimum cyclic and stripping analytical working range was 0.5–4.0 mL (3 × 10^2^~5 × 10^2^ CFU/0.5 mL) SA. The statistic relative standard deviation of 0.1 mL SA was observed to be 0.0078 (n = 5). Using the optimum parameters, a diagnostic test was performed by the direct assay of SA in non-treated human blood and patient sera. Here, the developed results can be used for the direct assay of non-treated blood sera, organ monitoring, in-vivo diagnosis, and other assays requiring SA detection.

## 1. Introduction

Staphylococcus aureus is a ubiquitous Gram-positive bacterium [1] and a food poisoning agent [2] that causes a variety of infections related to the human toxic shock syndrome and sudden infant death syndrome [3]. It can also cause serious infections, such as blood stream infections, pneumonia, or bone and joint infections. Here advanced diagnostic detection methods were recently developed, including one-step immune chromate graphic assay [4], gold nano particle based immune cromatographic assay [5], real-time nucleic-acid-sequence-based amplification assay [6], polymerase chain reaction (PCR) assay [7], real-time PCR assay [8], enzyme-linked immunosorbent assay [9], and multiplex PCR reversed passive latex agglutination (RPLA) identification [10]. Recently some of these basic methods depend on complicated PCR amplification, electric separation, spectric adsorption and other detection systems demand. However, in this study, simplified voltammetric methods are tested, which do not require complicated separation, expensive amplification or detection systems.

## 2. Objectives

In this study, an attempt was made to simplify the modification probe and to directly conduct non-treated blood assay for SA diseases. The assay is fast, sensitive, and does not require any preparation. Moreover, mercury immobilized [11] on a carbon nanotube structure [12] was used, where we sought a carbon surface that has catalytic effects [13], a large surface area, and high electrical conductivity [14], and whose probe can react with the SA target. Redox [15,16] voltammograms [17,18] were obtained via the square wave (SW) stripping and cyclic [19] voltammetric reaction [20,21]. The analytical parameters were optimized, and the results achieved low detection ranges for the SA target. The developed methods can be applied in the direct assay of non-treated blood sera, and the final results can be used for organ monitoring, in-vivo diagnosis, and other assays requiring SA detection.

## 3. Study Design

### 3.1. Instrument, Reagent and MCN Preparation

Diagnostic circuits were carried out using a BVA 2 voltammetric workstation from the authors’ institute. External electromagnetic noise was blocked by grounding with a Faraday iron box. The carbon nanotube probe (outside diameter: 15–40 nm; length: 30–50 um) was obtained from Nanotech Co., Ltd. (Seoul, Korea), Choong Nam 330–816, South Korea by chemical vapor deposition (CVD); it was purified overnight prior to use, via magnetic stirring in a 2 M nitric-acid solution, and was washed using triple-distilled pure water. SA was obtained from the Culture Collection and Research Center of biological bank of Korea. SA was maintained on tryptic soy agar slants, grown overnight on soy agar, and continually cultured at 37 °C, 24 ± 2 h until a concentration of 10^9^ colony-forming unit (CFU) mL^−1^ was reached (N = 6). It was diluted to 3 × 10^2^~5 × 10^2^ CFU/1 ml using 0.85% NaCl electrolyte. The MCN working electrode was made using paste consisting of a mixture of 40 wt% carbon nanotube, 40 wt% Hg (standard 1000 ppm from Sigma), and 20 wt% reagent-grade mineral oil (for conductivity and water proof, NJ, USA, 1-800-01, Acro). The mixed paste was inserted into a 3-mm-diameter, 100-mm-long catheter capillary and was stabilized with a 10-cycle scan from 1.0 V initial potential to −1.0 V switching potential, at a 0.5 V/s scan rate, in an electrolyte solution. Voltammetric assay was performed using the three-electrode system. A 3.0-mm-diameter graphite pencil electrode (GC) was prepared from common pencil lead (DongA XQ, ceramic, 60 mm, 0.9 B). A 1.0-mm-diameter, 10-mm-long platinum metal wire working electrode was made, and GC was used as an Ag/AgCl/KCl reference and platinum counter electrode. This three electrode system was immersed in a solution of 1.0-mL non-treated human blood serum as an electrolyte solution. All the experiments were performed at room temperature, without removing the oxygen. Human blood and patient sera were obtained from the National Blood Transfusion Research Institute.

### 3.2. Cyclic Properties of the GC and MCN

**Figure 1:**
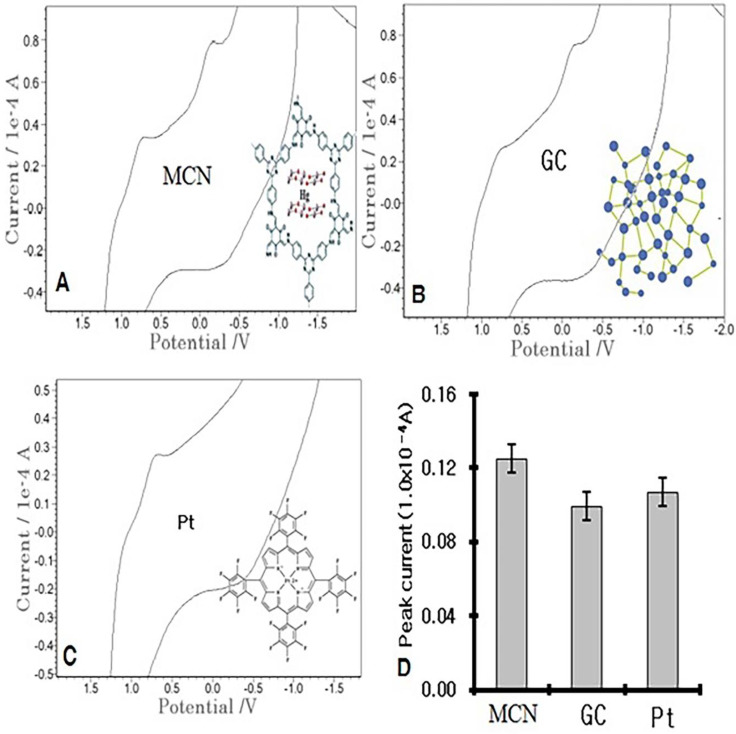
Cyclic voltammograms of the MCN (**A**), GC (**B**), and Pt (**C**) probes in an SV 1.0 × 10^−^³ L patient serum, with 30 s accumulation time, −2.0 V initial potential, 2.0 V switching potential, 0.1 V/s scan rate, and the ionic oxidation peak current (**D**).

Voltammetric reaction potentials depend on the anodic and cathodic adsorption electro transfer activities. Therefore, the peak potential was sought in the 1.0-mL patient serum electrolyte. Three-electrode systems and the prepared sensor probes were directly inserted, after which multifid scanning was performed on the common-type GC and metal Pt, and the MCN probes were compared using cyclic redox scan. Figure 1 shows the real voltammograms of the common-type GC, Pt, and specified MCN probes, using the same blood sera and identical parameters. In the anodic scan, only probe oxidation with no signals appeared, but in the redox scan, two peaks appeared at 0.8 and −0.2 V only in the MCN and GC electrodes, and Pt had only one peak. The peak currents are shown in the figure. The peaks were 0.126 × 10^−4^ A MCN, 0.0992 × 10^−4^ A GC, and 0.107 × 10^−4^ A Pt. The immobilized-mercury effect is more sensitive than that of the common-type electrode. The results of this study can be applied to stripping voltammetry; thus, using the same cell systems, stripping voltammetric scan was performed on anodic and cathodic activities, and the parameters that were used were −2.0 V initial potential, 2.0 V final potential, and 30 s accumulation time. Each voltammogram was obtained only for 0.047 × 10^−4^ MCN, 0.059 × 10^−4^ A GC, and 0.05 × 10^−4^ Pt anodic, and not for the cathodic results. Moreover, the study results indicate that MGC is more sensitive than Pt and GC (results not shown). The peak potential was used for the diagnostic assay of SA for the human blood, then more expanded analytical properties were examined using square-wave stripping voltammetry.

## 4. Results

### 4.1. Stripping Voltammetric SA and Positive Patient Sera Using MCN

**Figure 2:**
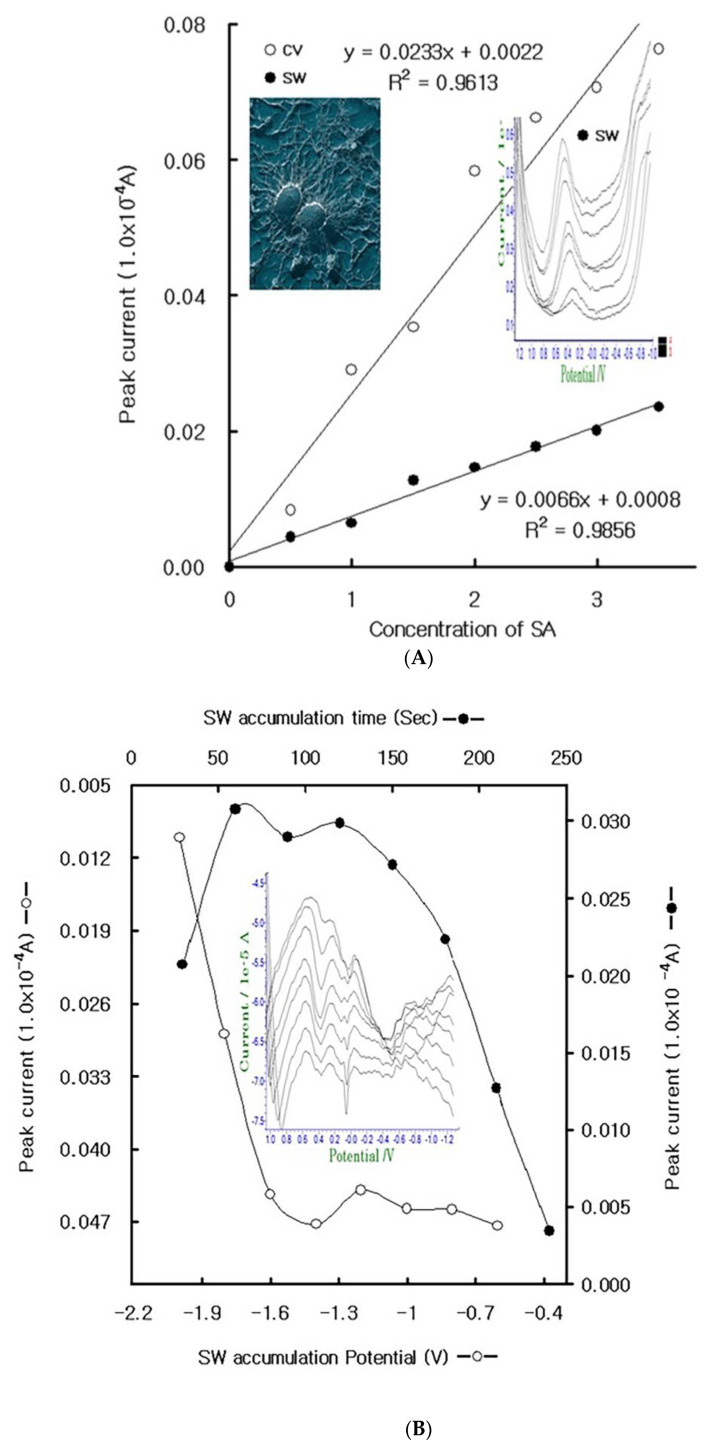

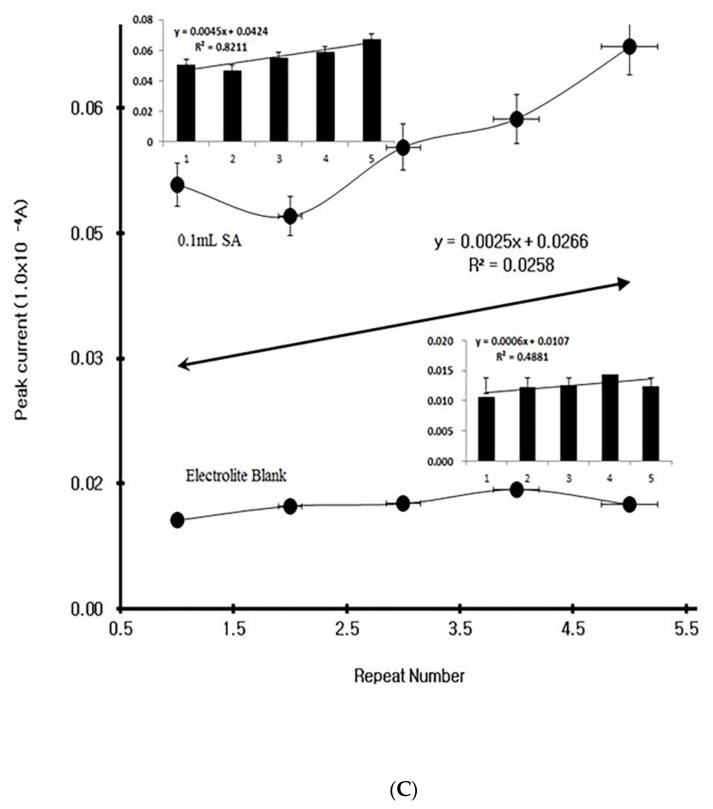
(**A**) Analytical working variations for the 0.5-, 1.0-, 1.5-, 2.0-, 2.5-, 3.0-, and 3.5-ul SA add in 1-mL serum using the MCN working electrode for CV -○- and SW -●-. (**B**) SW accumulation time variation for 30, 60, 80, 120, 150, 180, 210, and 240 s -●-, and SW accumulation potential variation for −2.0, −1.8, −1,6, −1.4, −1.2, −1.0, −0.8, and −0.6 V -○-. (**C**) Statistic MCN probe stability in serum blank and SA spike. Other parameters were used for the optimum conditions.

Under optimum conditions, the diagnostic working ranges were examined using SA spiking in the healthy sera. In the 1.0 × 10 ^−^³ L healthy plasma, sequential addition was performed with 240 s accumulation stripping time. Figure 2 shows the voltammetric results.

Under MCN probe, Figure 2A shows the voltammetric anodic peak current for the cyclic (white curve) and stripping (black curve) voltammetric anodic scan. Here, a cyclic peak of 0.29 × 10^−4^ A appeared at the 0.5 V reduction potential, which later increased to 0.076×10^−4^ A, where the linear curve was y = 0.0233x + 0.00220 and R^2^ = 0.9613, whose slope is more sensitive than that of stripping voltammetry. Moreover, the stripping slope was △x/△y = 0.0066, the intercept was 0.0008, and the statistic was R^2^ = 0.9856. Both equations can be used for diagnostics. Thus, the more sensitive parameters of the accumulation effects were examined using MCN, and Figure 2B shows the results, as follows: anodic peak high for time variation (-●-), 30–240 s; accumulation potential variation effects (-○-), −2.0 to −0.6 V. Peak currents of 0.007–0.047 × 10^−4^ A were obtained, and 240 s was the maximum accumulation time. Moreover, the stripping potential varied from 0.0038 to 0.0289×10^−4^ A, and −2.0 V was the maximum stripping potential. Thus, the 240 s accumulation time and −2.0 V stripping potential were fixed. Under these conditions, the probe stability was examined via repeated stripping. Figure 2C shows the peak currents for the sera blank and 0.1-mL SA spiked voltammograms. The blank current varied from 0.014 to 0.011 × 10^−4^ A, and the 0.1-mL SA spikes were 0.047–0.067 × 10^−4^ A. MCN is thus stable and can be used for diagnostics, with the final parameters fixed at −2.0 V accumulation potential, 0.02 V amplitude, 25 Hz frequency, 0.01 V incremental potential, and 240 s accumulation time, using the conditions under which the analytical working ranges were examined.

### 4.2. Diagnostic Working Ranges of SA

**Figure 3:**
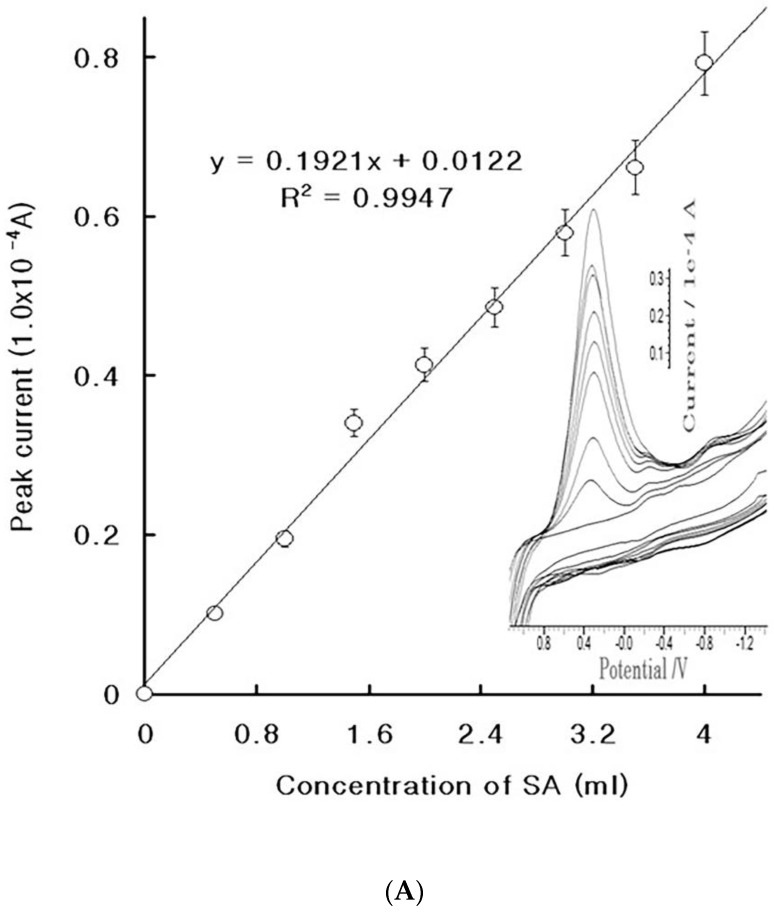

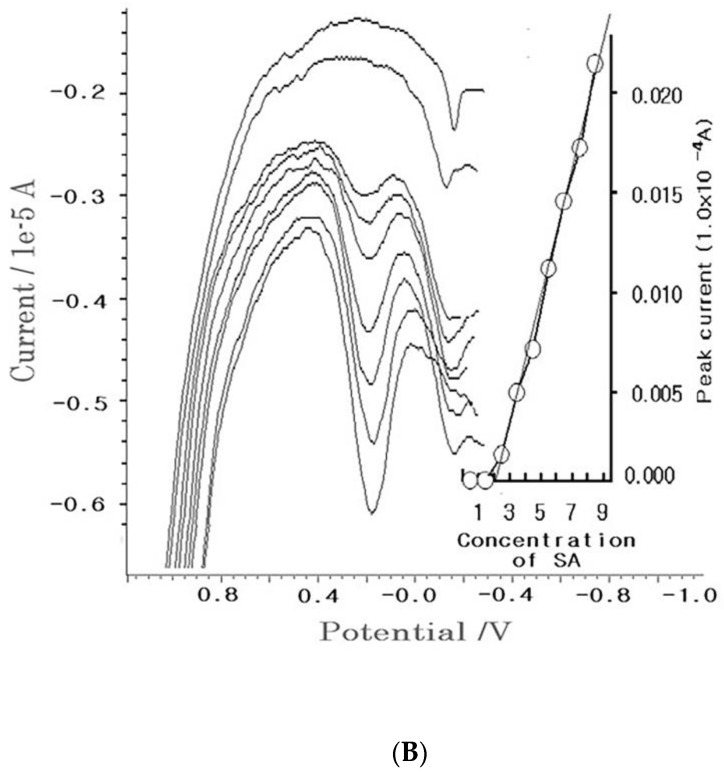
(**A**) Cyclic linear voltammograms for the 0 × 10^−^³, 0.5 × 10^−^³, 1.0 × 10^−^³, 1.5 × 10^−^³, 2.0 × 10^−^³, 2.5 × 10^−^³, 3.0 × 10^−^³, 3.5 × 10^−^³, and 4.0 × 10^−^³ L SA spikes. (**B**) Cathodic stripping voltametric working ranges of the 0.5 × 10^−^³, 1.0 × 10^−^³, 1.5 × 10^−^³, 2.0 × 10^−^³, 2.5 × 10^−^³, 3.0 × 10^−^³, 3.5 × 10^−^³, and 4.0 × 10^−^³ L SA spikes in a 1.0 × 10^−^³ L non-treated blood serum with a pH of 7.0 and 0.02 V SW amplitude, 75 Hz SW frequency, 0.01 V increment potential, −2.0 V accumulation potential, and 90 s (CV) and 210 s (SW) accumulation times. The optimum conditions were set for the other parameters.

Under CV conditions, the linear working voltammetrics were sought at the 0.5- to 4.0-mL spikes. The reduction peak is shown in Figure 3A. Only a 0.2 V reduction peak appeared, and the first peak was that of the electrolyte blood serum. There were no signals at the 0.5-mL spike, then a peak of 0.0013 × 10^−4^ A was obtained at the 1-mL spike, with 0.2 V reduction potential, which continually increased to a peak of 0.7921 × 10^−4^ A at the 4-ml spike, with a slop of dx/dy = 0.192, an intercept of 0.012, and a relative statistic of 0.9947. The error percentage is shown on this curve. These results can be applied to diagnostics. Moreover, at the same spike range, a linear curve was obtained at seven points. Figure 3B shows the real voltammograms, where the first curve (for the electrolyte blood serum) is simple, then very small peaks were obtained at the 0.5- and 1.0-mL spikes, after which a 0.0044 × 10^−4^ A peak was obtained at the 1.5-mL spike. Moreover, at the 4-mL spike, a peak of 0.021 × 10^−4^ A was obtained, whose final curve was y = 0.005x − 0.002 and whose statistic was R^2^ = 0.9649. Under these conditions, the accumulation times that were used were 90 and 210 s for CV and SW stripping accumulation, respectively. As the SW effects were found to be much more sensitive than the CV effects, the former can be used for diagnostics. Under these conditions, the interference effects were calibrated using the standard addition methods, after which diagnostics was applied to the patients’ sera.

### 4.3. Patient Diagnosis

**Figure 4:**
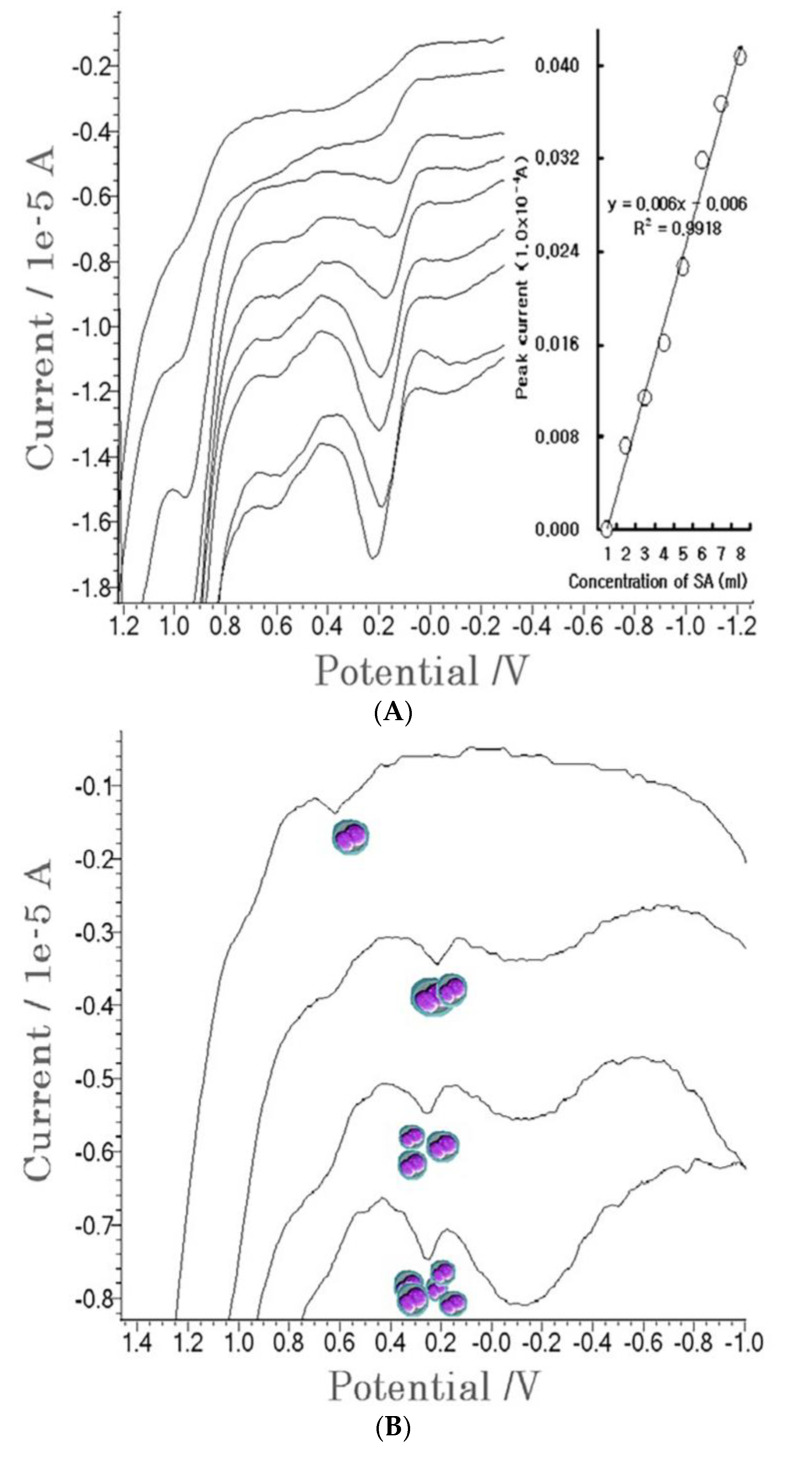
Positive patient test. (**A**) Anodic stripping for the 1.0 × 10^−^³ L patient sera, where the stripping conditions were 25 Hz frequency, 20 mV amplitude, −2.0 V accumulation potential, 100 s accumulation time, and 20 × 10^−^³ V increment potential. (**B**) The standard addition methods used on the patients’ red cells, using the optimum SW conditions.

Under a sera solution, diagnostic application was performed on the patient plasma, via SW anodic stripping voltammetry. The contaminated serum sample was obtained from the National Blood Transfusion Research Institute. Figure 4A shows real voltammograms for 1.0-mL blood testing. The first curve represents the contaminated blood. In the 0.2 V anodic scan, a small peak current of 0.0072 × 10^−4^ A was obtained, using 90 s accumulation stripping, then standard SA was spiked to 0.5, 1.0, 1.5, 2.0, 2.5, 3.0, and 3.5 mL. The peak current linearly increased from 0.0113 to 0.0408 × 10^−4^ A. The regression equation was y = 0.006x − 0.006, and the relative standard deviation was R^2^ = 0.9918, which could be detected for 6.0-ul SA. Then more advanced tests were performed using a red corpuscle. Figure 4B shows the results of the use of the standard addition methods, where the first curve represents the red blood cells using optimum parameters. A peak current of 0.0009 × 10^−4^ A was obtained, and 0.0021–0.0055 × 10^−4^ A peak currents were obtained at the 1-, 2-, and 3-mL SA spikes. The working equation was y = 0.0016x − 0.0008, the statistic was R^2^ = 0.9922, and the content was 0.8ul. The results of the study can be used for diagnostics in any application.

## 5. Conclusions

A novel SA bioprocess was developed using an MCN sensor in non-treated human blood and patient sera. The immobilized-mercury effects were found to be more sensitive than those of the common probe type using cyclic voltammetry, where the following optimum analytical parameters were obtained: −2.0 V accumulation potential, 0.02 V amplitude, 25 Hz frequency, 0.01 V incremental potential, and 240 s accumulation time. Under these conditions, the standard deviation of the 0.1-m (15 × 10^2^~25 × 10^2^ CFU) SA was 0.0078%. This result was more sensitive and precise than other existing methods [21,22,23]. The results of this study can be applied to a diagnostic assay for positive patient sera and SA infections, and can be used in real-time medical diagnosis as well as in direct in-vivo monitoring.

## Data Availability

All the original data in this article are from Biosensor Research Institute.

## Abbreviations

SA	Staphylococcus aureus
MCN	mercury immobilized on a carbon nanotube sensor
SW	stripping voltammetry
PCR	polymerase chain reaction
GC	graphite pencil electrode
RPLA	reversed passive latex agglutination
CFU	colony-forming unit
CVD	chemical vapor deposition
CV	Cyclic voltammetry
